# Supplementary material for: Gene Expression Divergence Between Locally Adapted Inland Annual and Coastal Perennial Ecotypes of Mimulus guttatus Across Developmental Stages
Source: Mol Ecol. 2025 Aug 14;34(22):e70080. doi: 10.1111/mec.70080 (PMC12617065; doi:10.1111/mec.70080)
Supplement: Supplementary file 1 — Figures S1‐S27: mec70080‐0001‐Figures.pdf. [file MEC-34-e70080-s002.pdf]

Supplementary Figures and Tables

Gene expression divergence between locally adapted inland annual and coastal perennial ecotypes of *Mimulus guttatus* across developmental stages

Jason D. Olsen[a,b], Billie A Gould[a], Yani Chen[a],and David B. Lowry[a,b,c,*]

[a]Department of Plant Biology, Michigan State University, East Lansing, MI, USA 48824
[b]Ecology, Evolution, and Behavior, Michigan State University, East Lansing, MI, USA 48824
[c]Plant Resilience Institute, Michigan State University, East Lansing, MI, USA 48824
*Correspondence should be addressed to D.B.L. (dlowry@msu.edu)

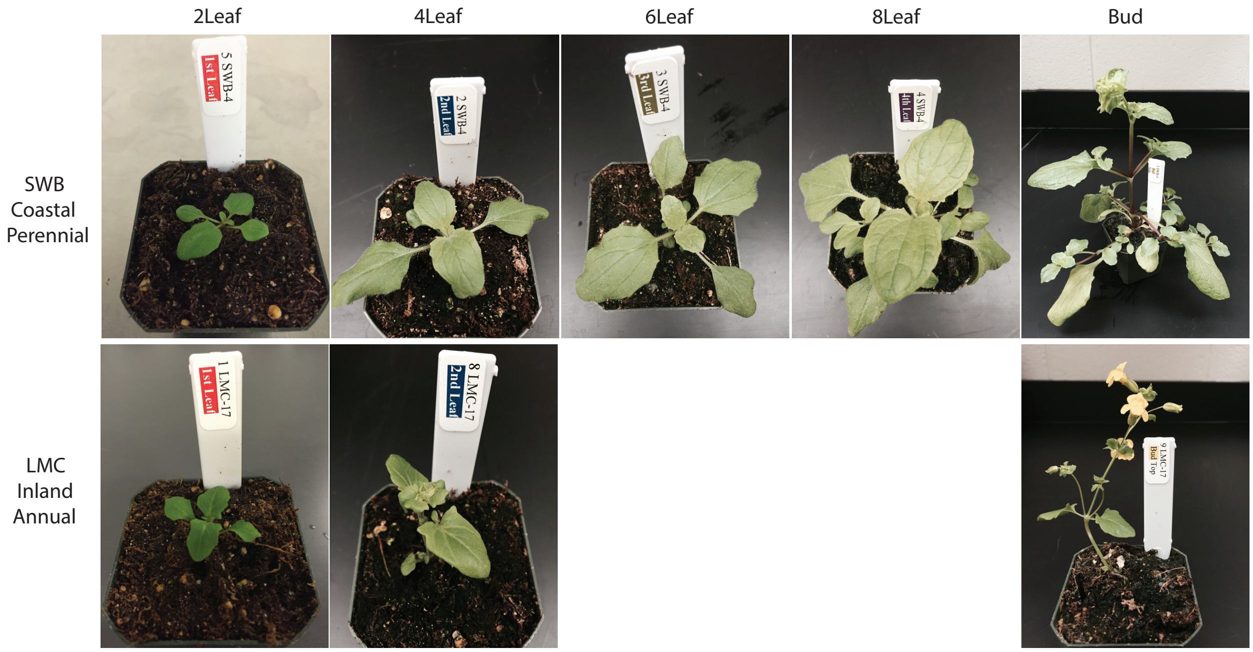

Figure S1. Photos of experimental plants on the day of tissue collection for RNA-seq through developmental stages for the two genotypes.

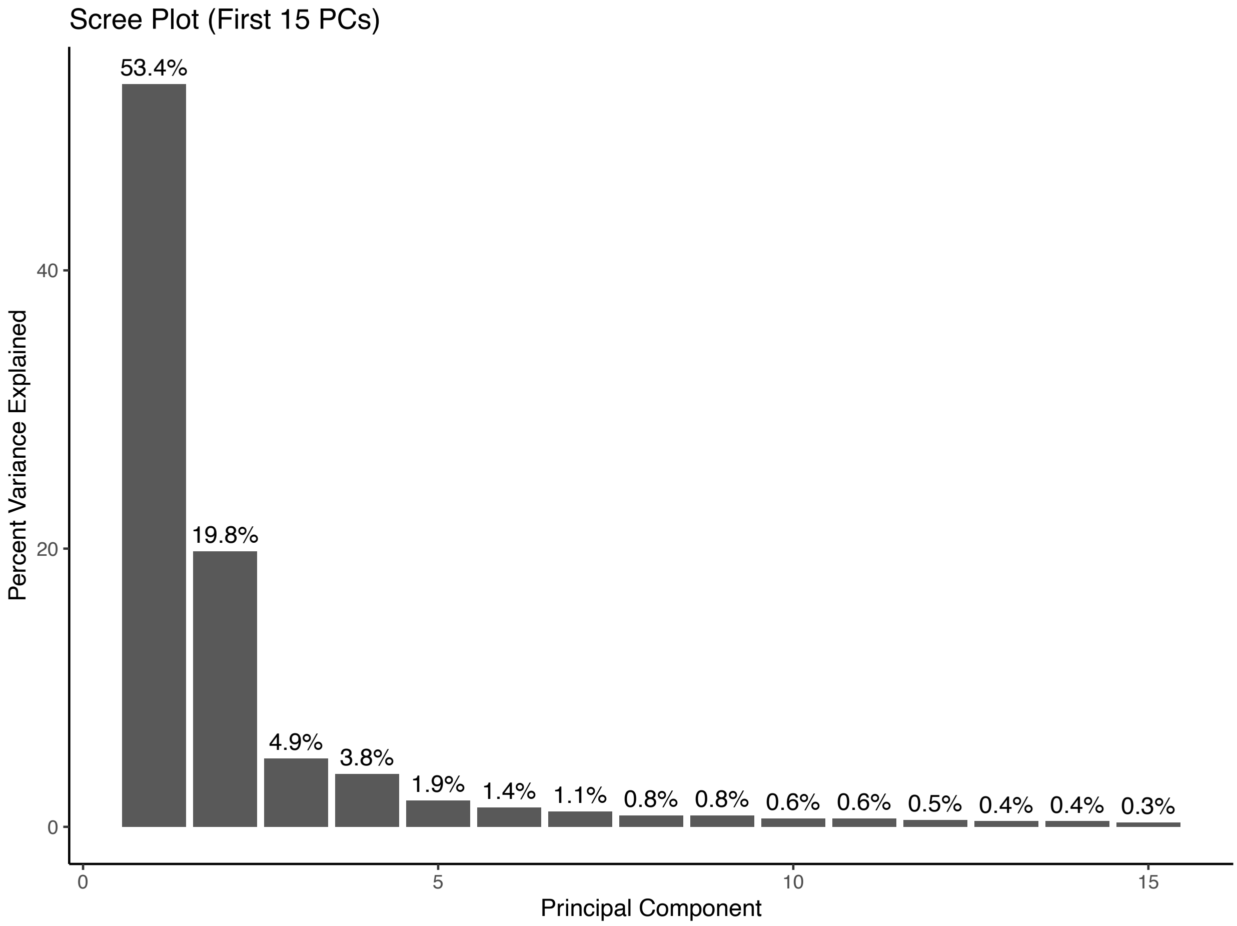

Figure S2. Scree plot of variance explained by the first 15 PCs for the principal component analysis (PCA) performed on variance stabilizing transformed (VST), pre-filtered read count data.

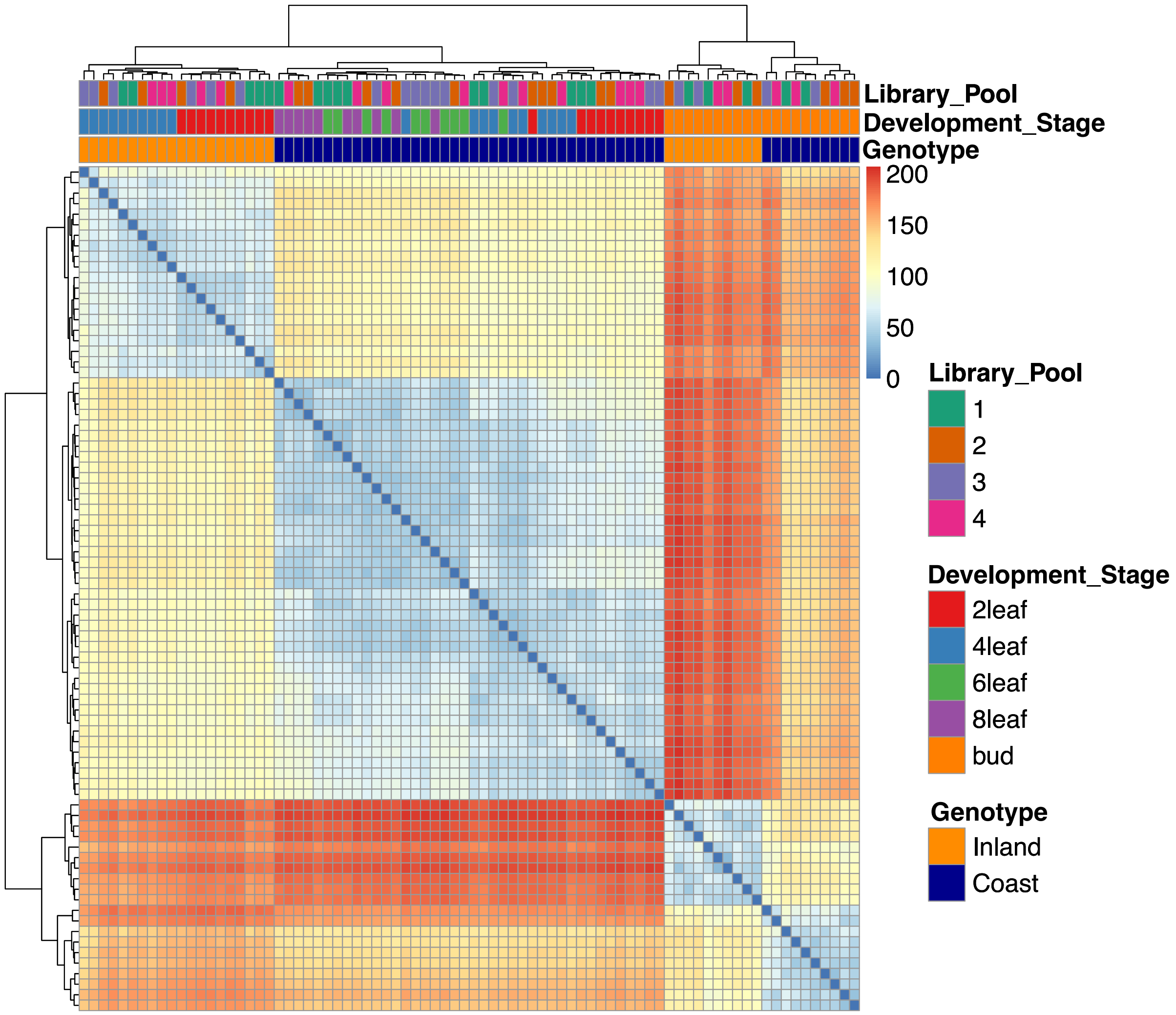

Figure S3. Heat map clustering of the 80 RNA-seq samples by genotype and developmental stage. There was no clustering by the four library pools, across which the samples were randomized.

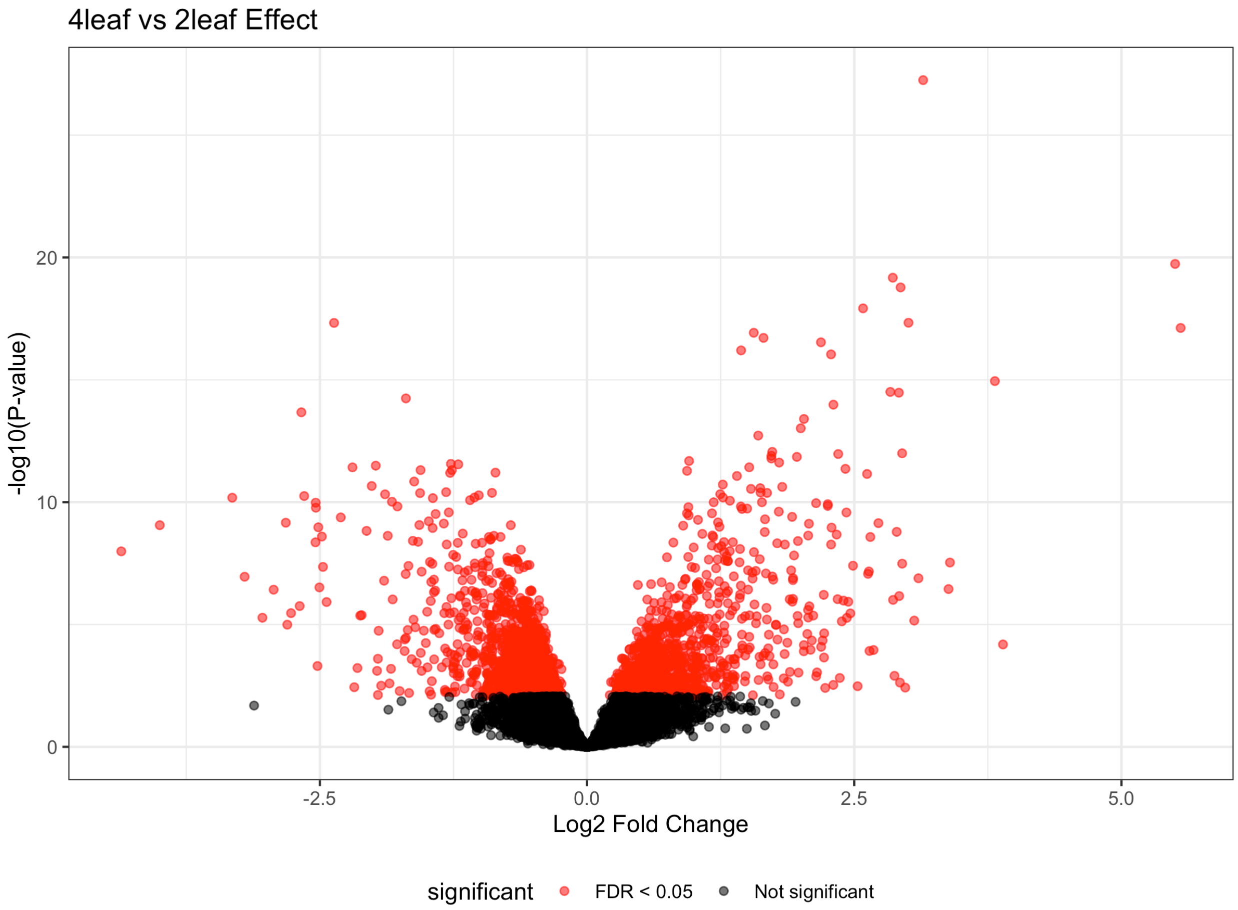

Figure S4. Volcano plot depicting the distribution of significantly differentially expressed genes (red) for the contrast between the second and fourth set of true leaves.

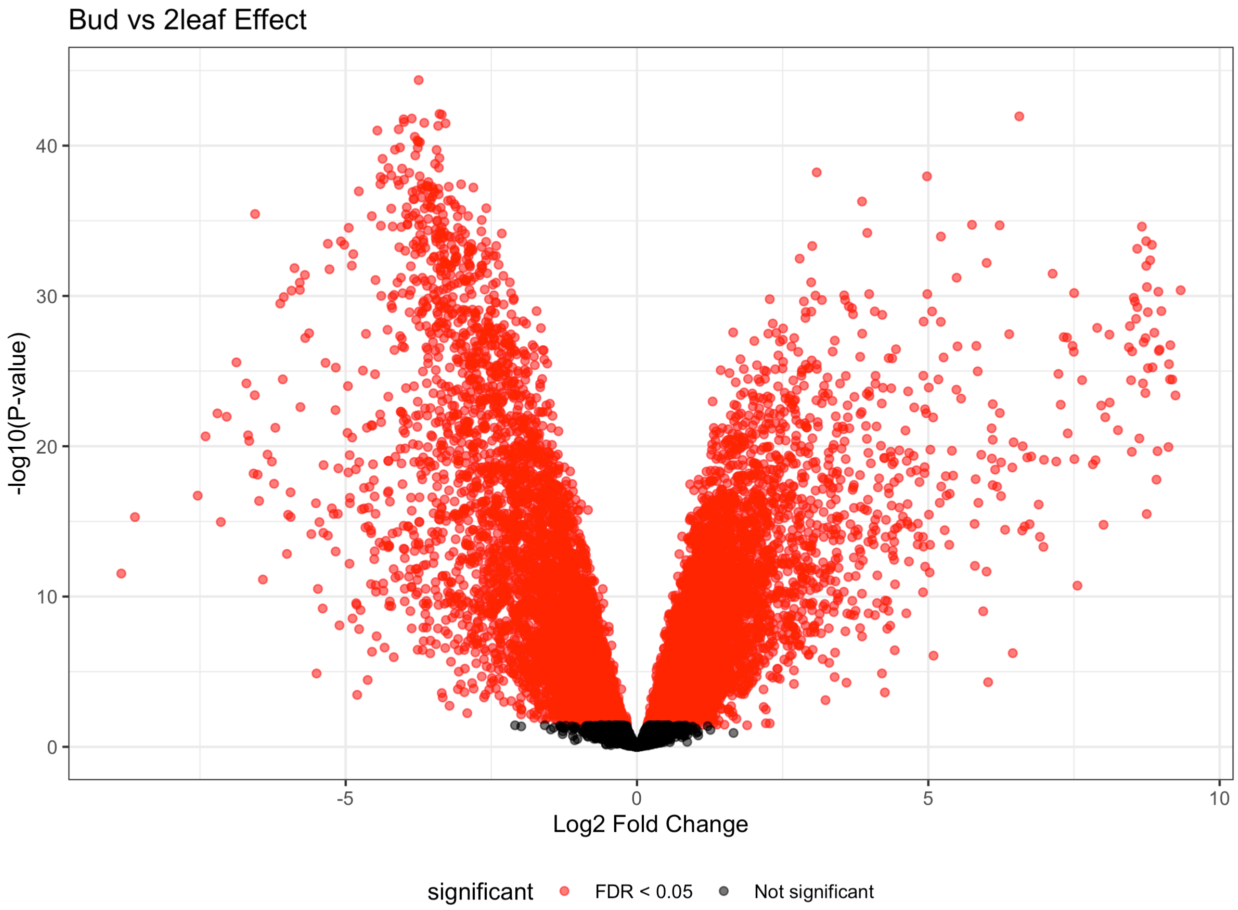

Figure S5. Volcano plot depicting the distribution of significantly differentially expressed genes (red) for the contrast between floral buds and the second set of true leaves.

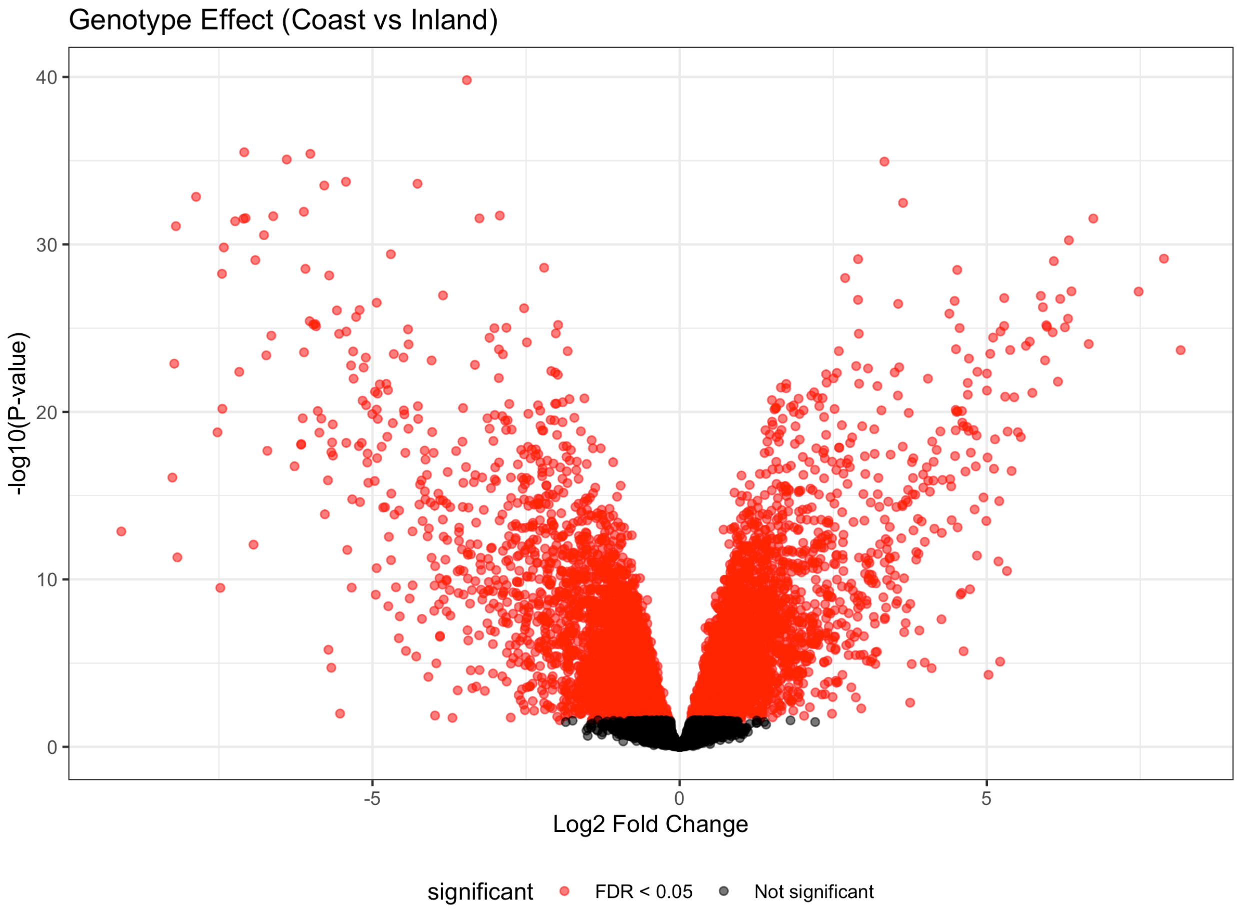

Figure S6. Volcano plot depicting the distribution of significantly differentially expressed genes (red) for the contrast between coastal perennial and inland annuall samples.

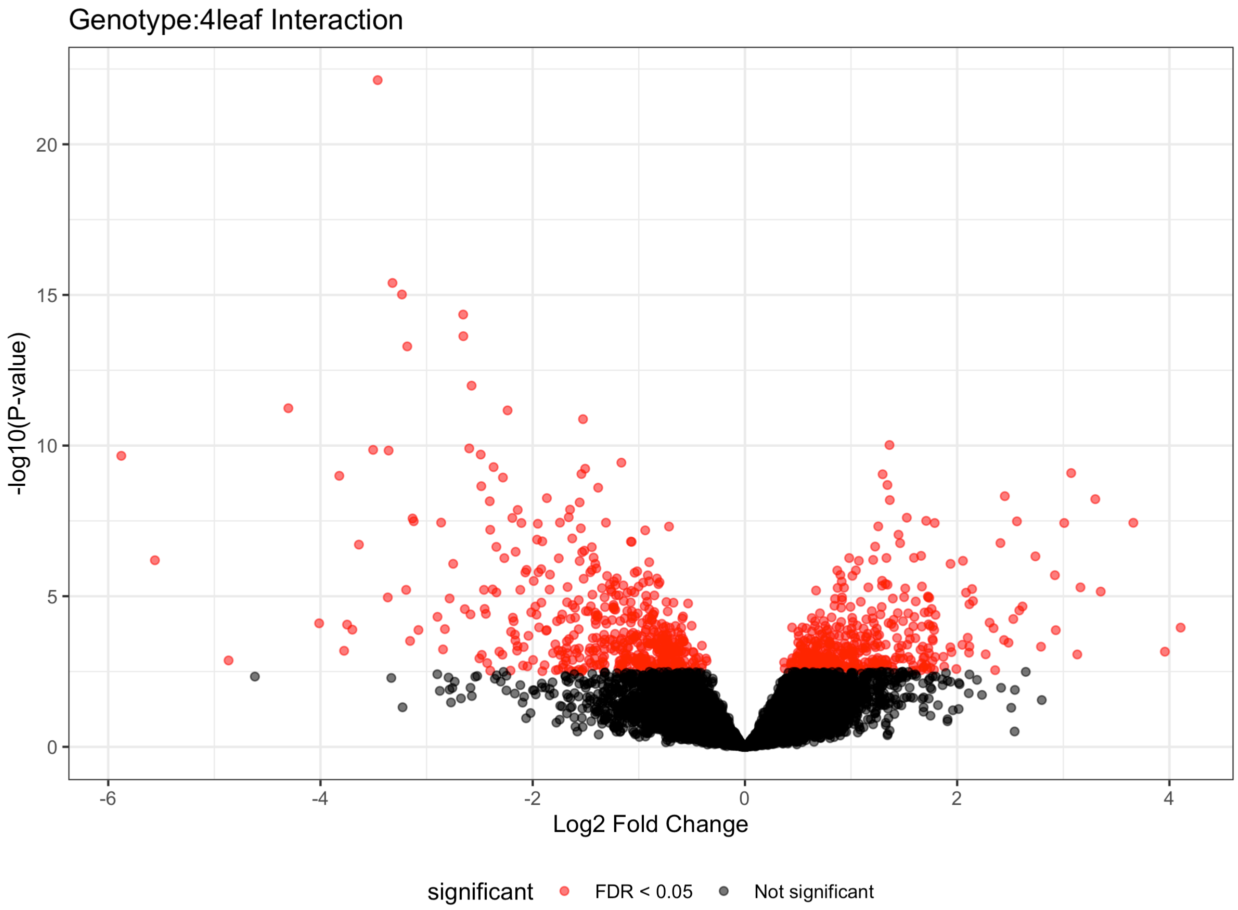

Figure S7. Volcano plot depicting the distribution of significantly differentially expressed genes (red) for the interaction of genotype with the contrast of the second and fourth set of true leaves.

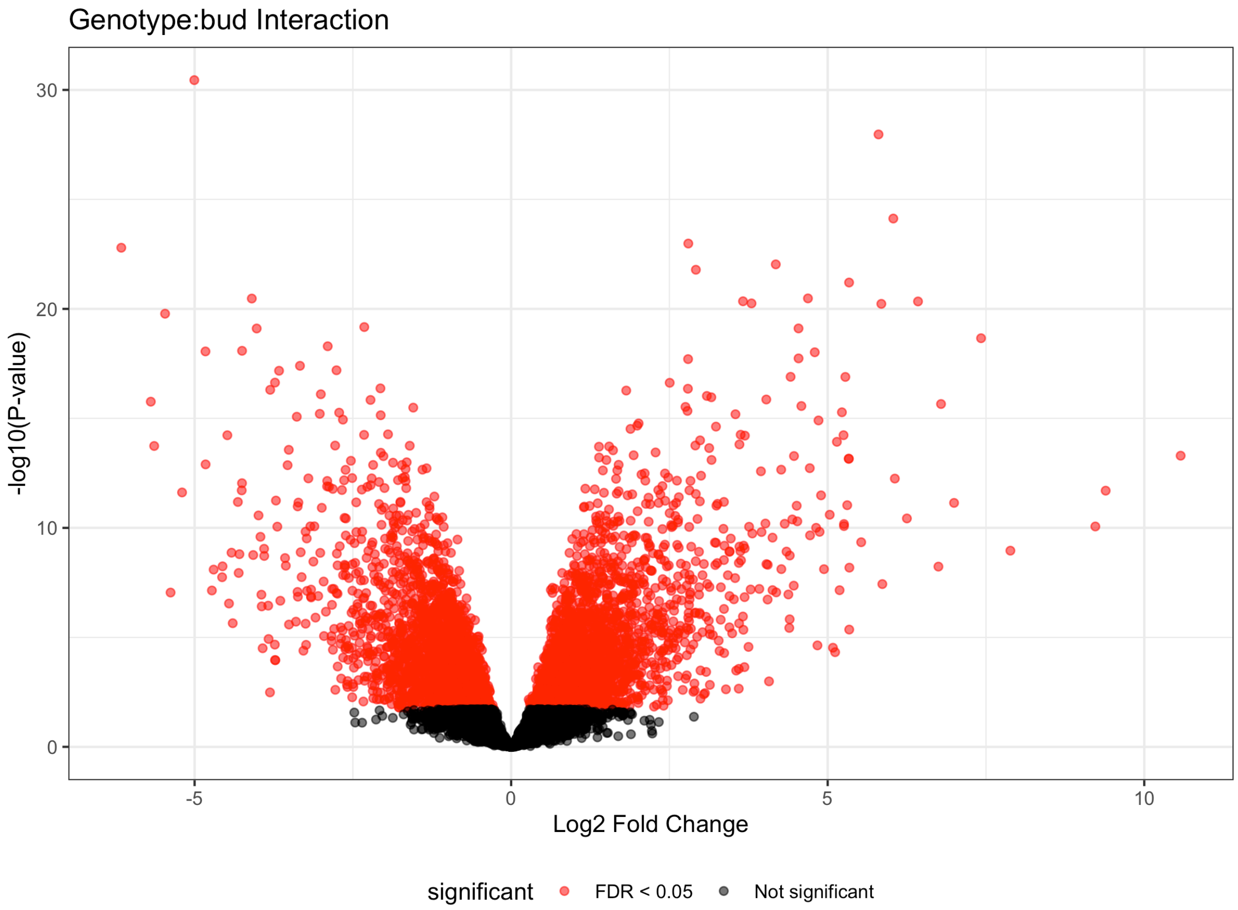

Figure S8. Volcano plot depicting the distribution of significantly differentially expressed genes (red) for the interaction of genotype with the contrast of floral bud and the second set of true leaves.

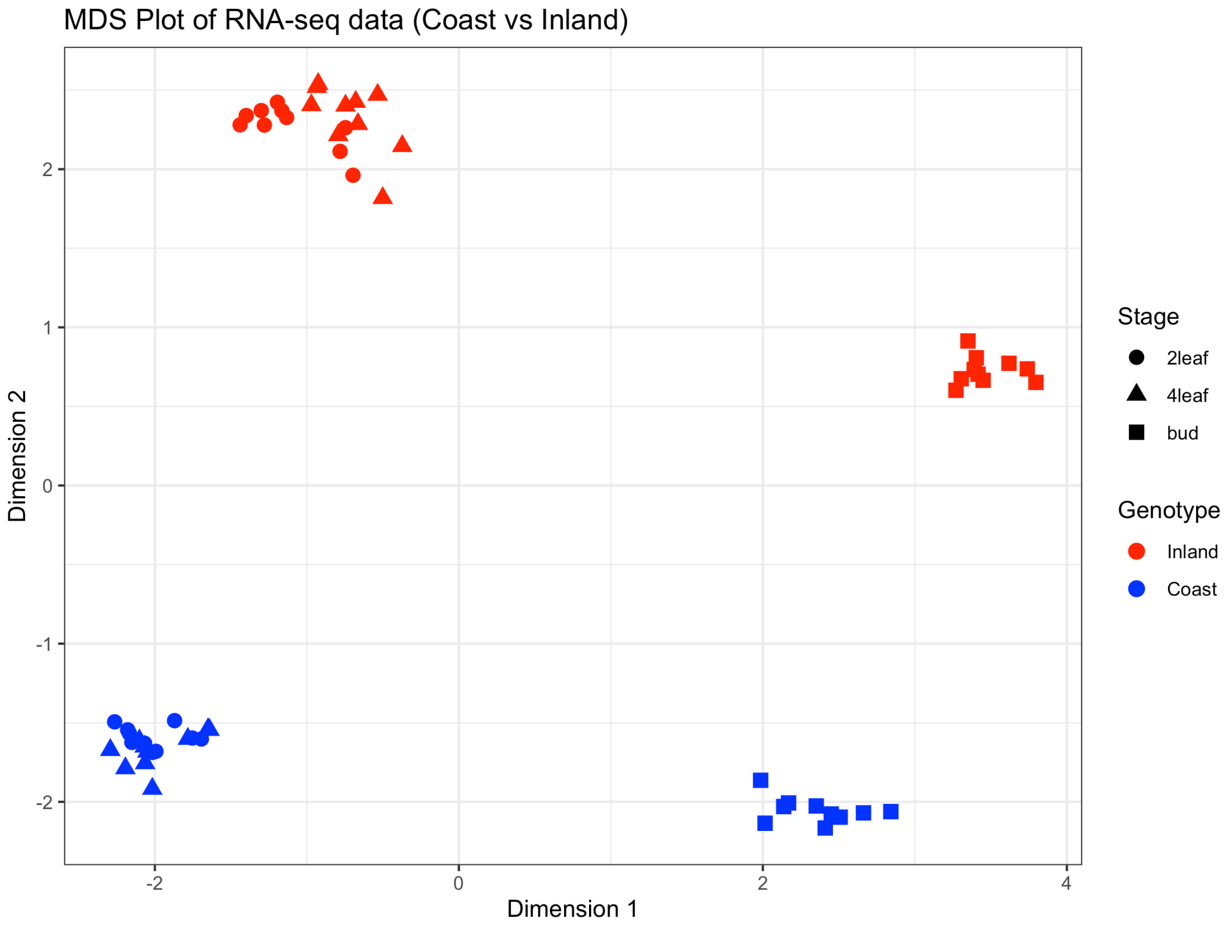

Figure S9. Multidimensional scaling (MDS) plot of the relationship of samples based on the 13,920 genes individually evaluated for differential expression using the voom-limma approach.

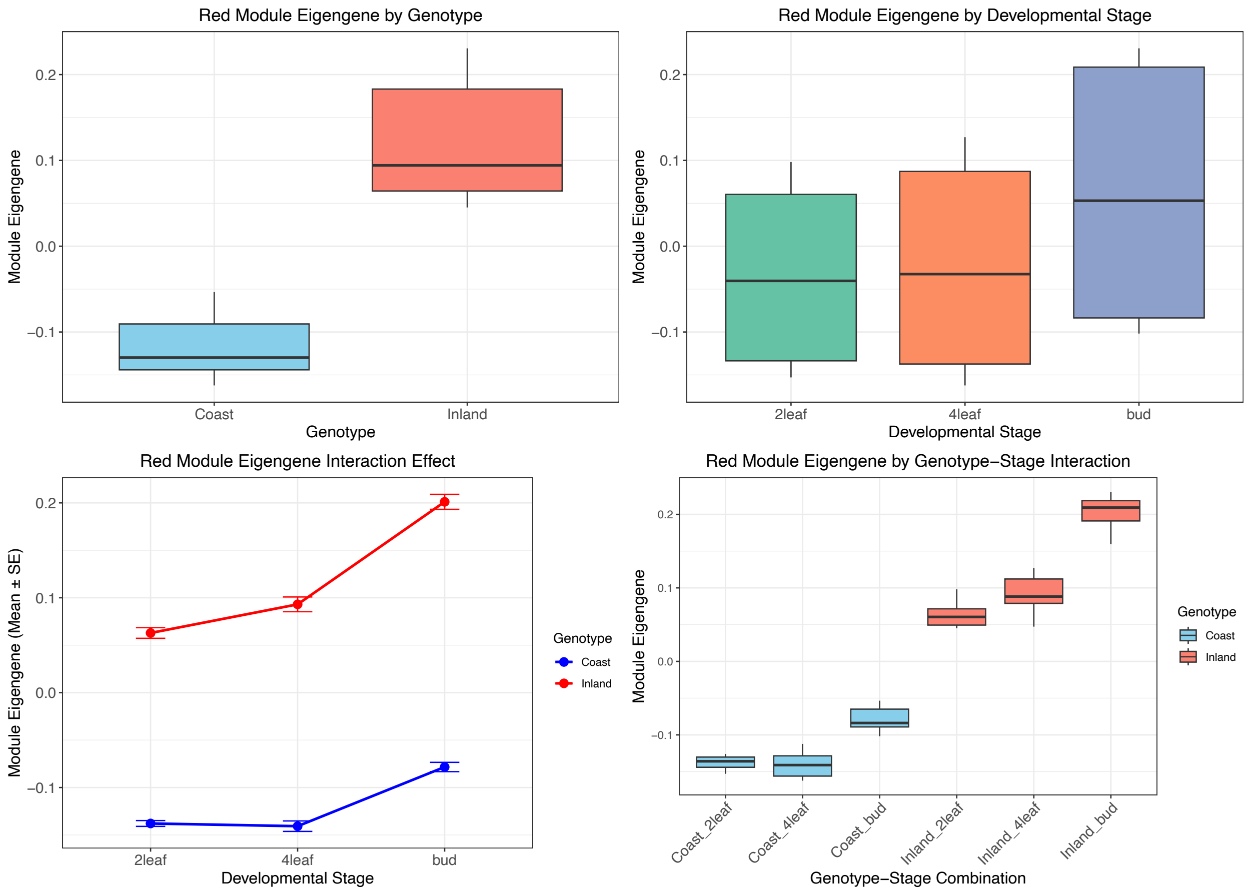

Figure S10. The main effects (genotype and developmental stage) and interactions for the eigengene of the red module from the WGCNA analysis.

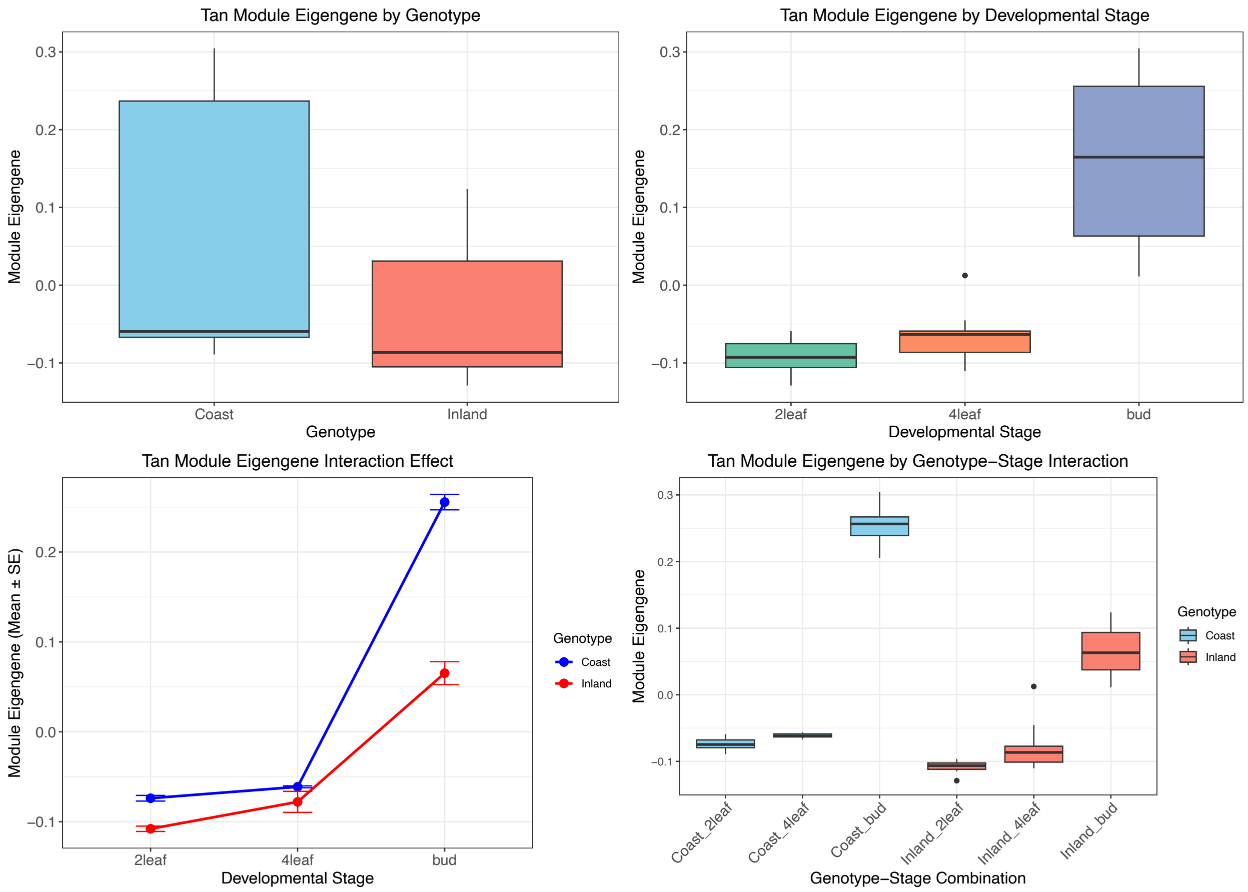

Figure S11. The main effects (genotype and developmental stage) and interactions for the eigengene of the tan module from the WGCNA analysis.

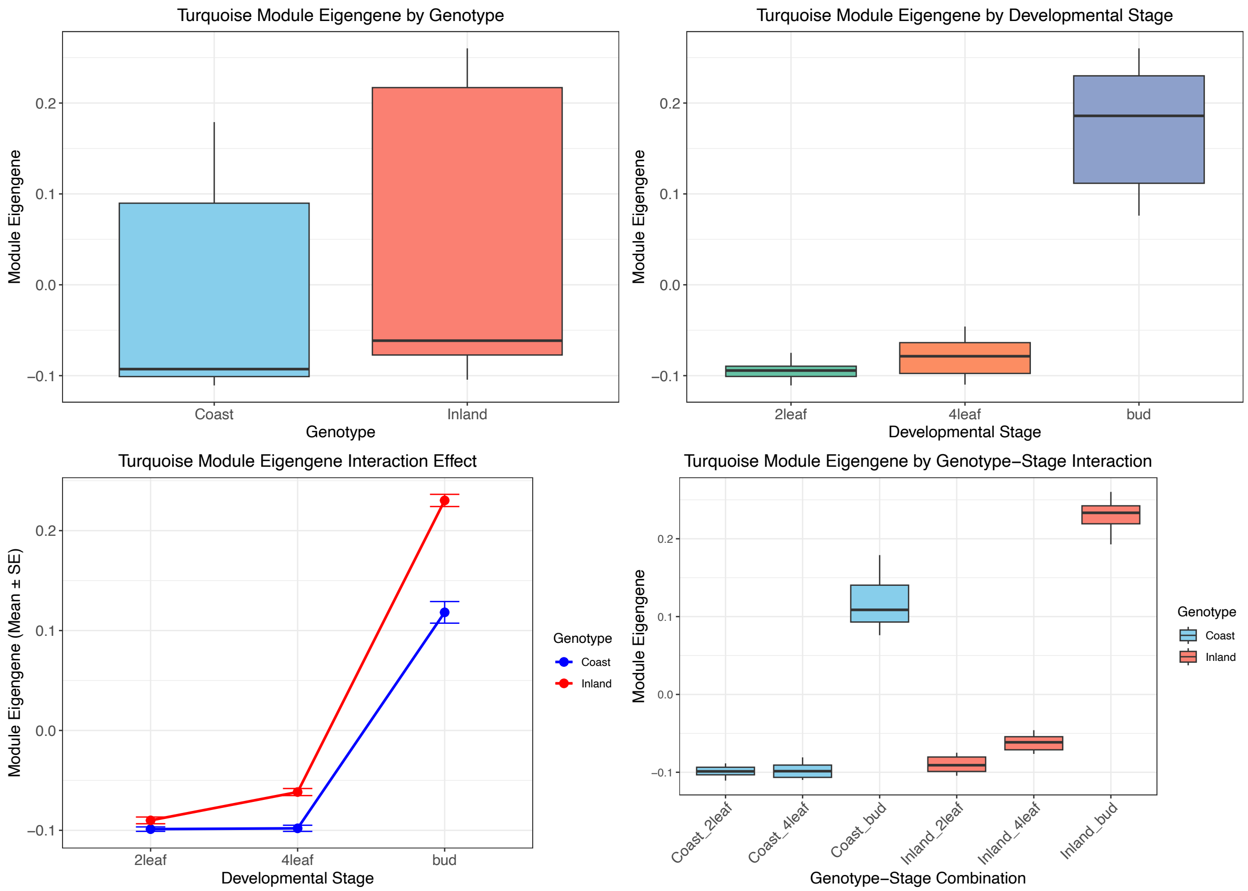

Figure S12. The main effects (genotype and developmental stage) and interactions for the eigengene of the turquoise module from the WGCNA analysis.

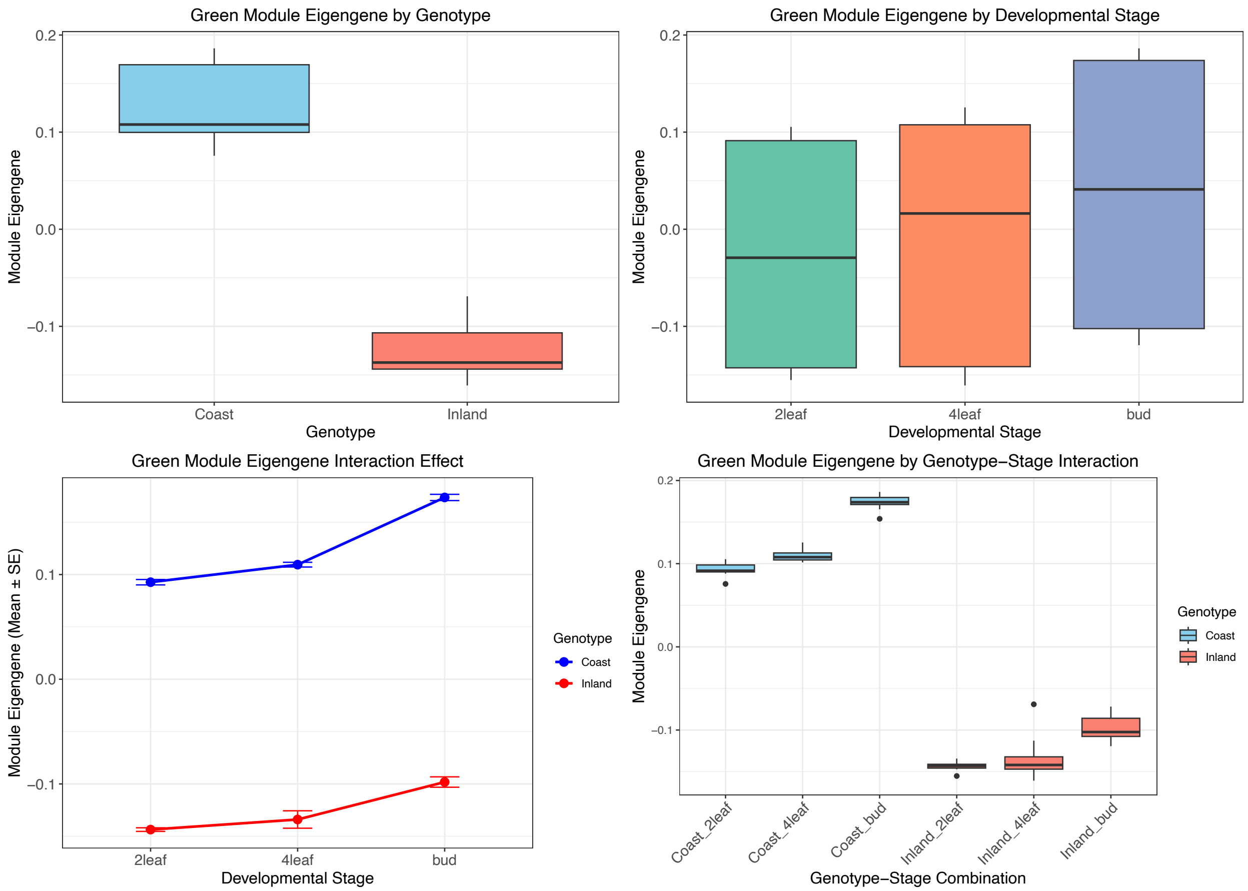

Figure S13. The main effects (genotype and developmental stage) and interactions for the eigengene of the green module from the WGCNA analysis.

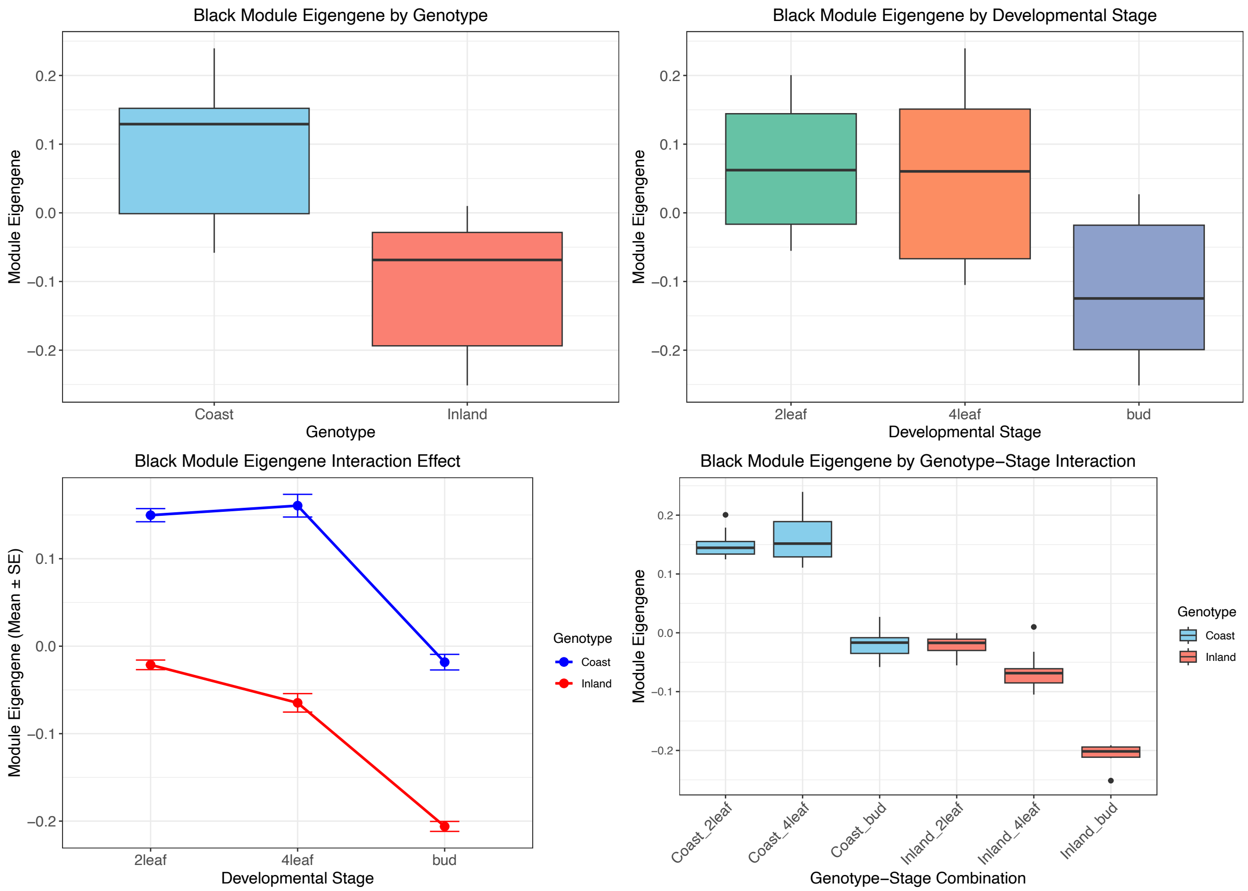

Figure S14. The main effects (genotype and developmental stage) and interactions for the eigengene of the black module from the WGCNA analysis.

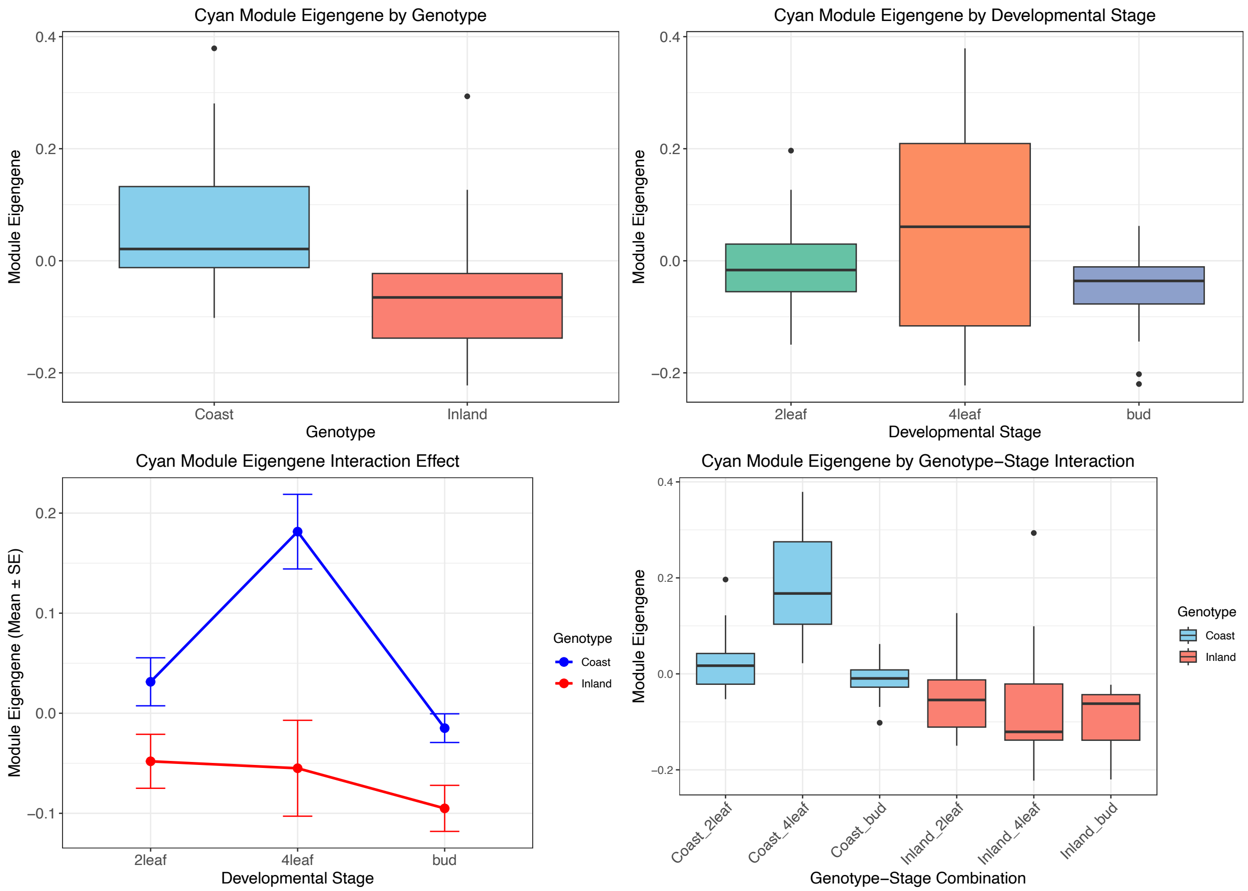

Figure S15. The main effects (genotype and developmental stage) and interactions for the eigengene of the cyan module from the WGCNA analysis.

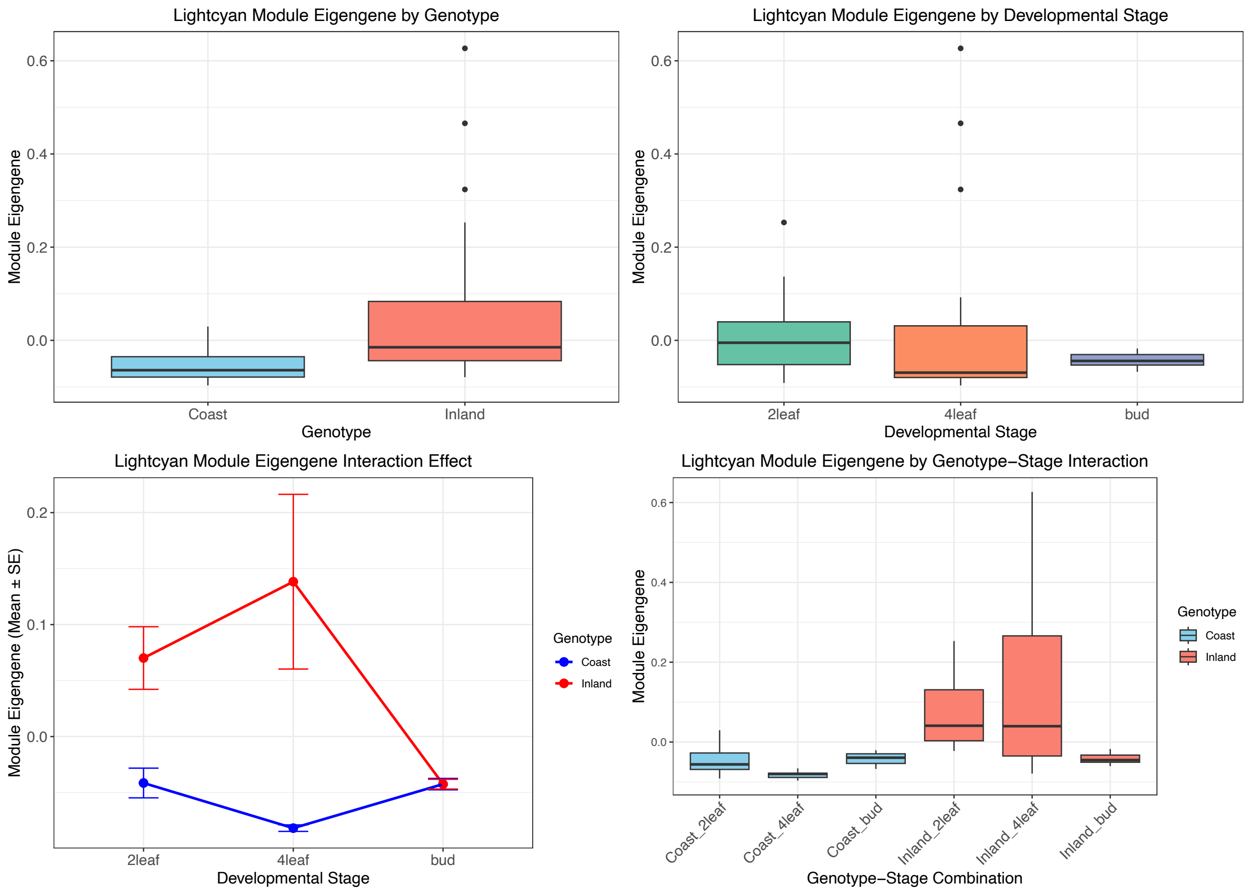

Figure S16. The main effects (genotype and developmental stage) and interactions for the eigengene of the lightcyan module from the WGCNA analysis.

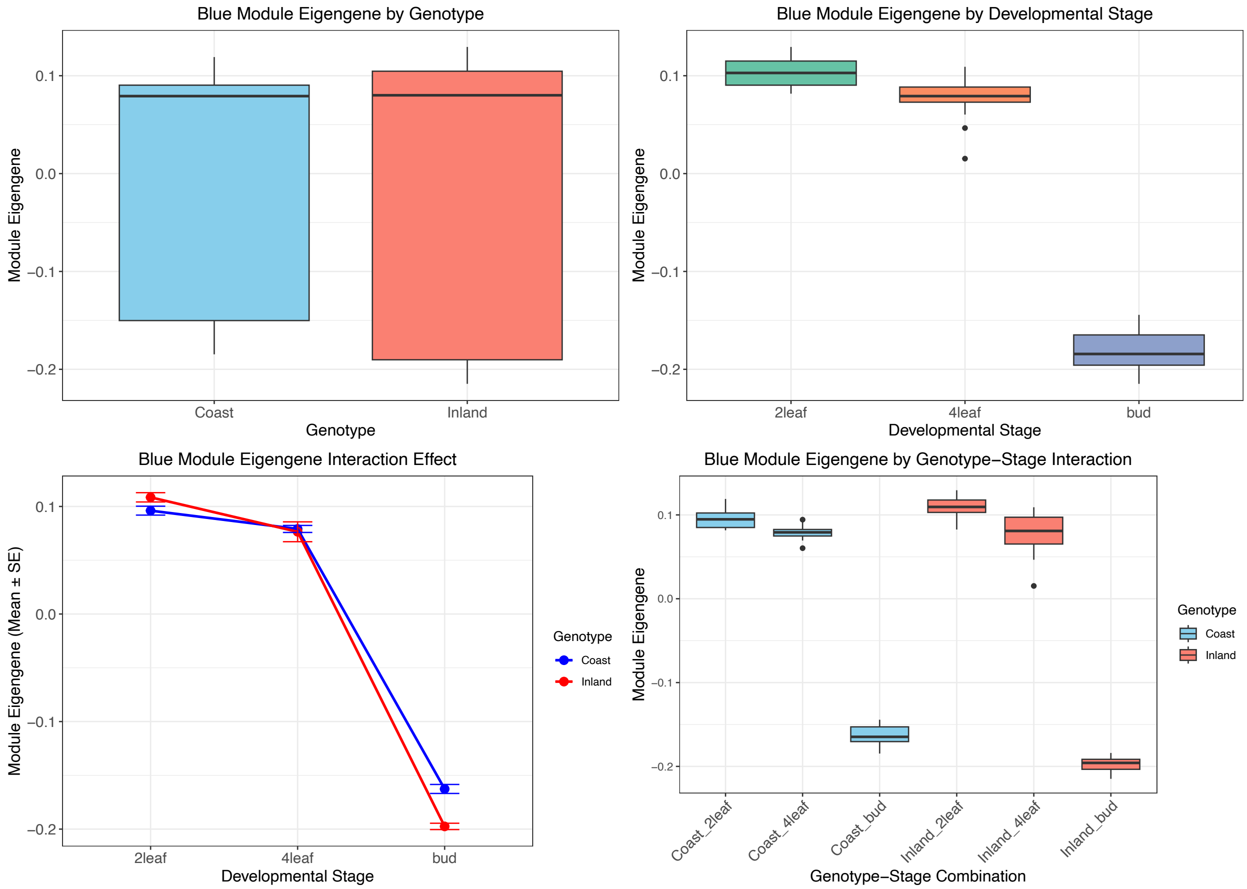

Figure S17. The main effects (genotype and developmental stage) and interactions for the eigengene of the blue module from the WGCNA analysis.

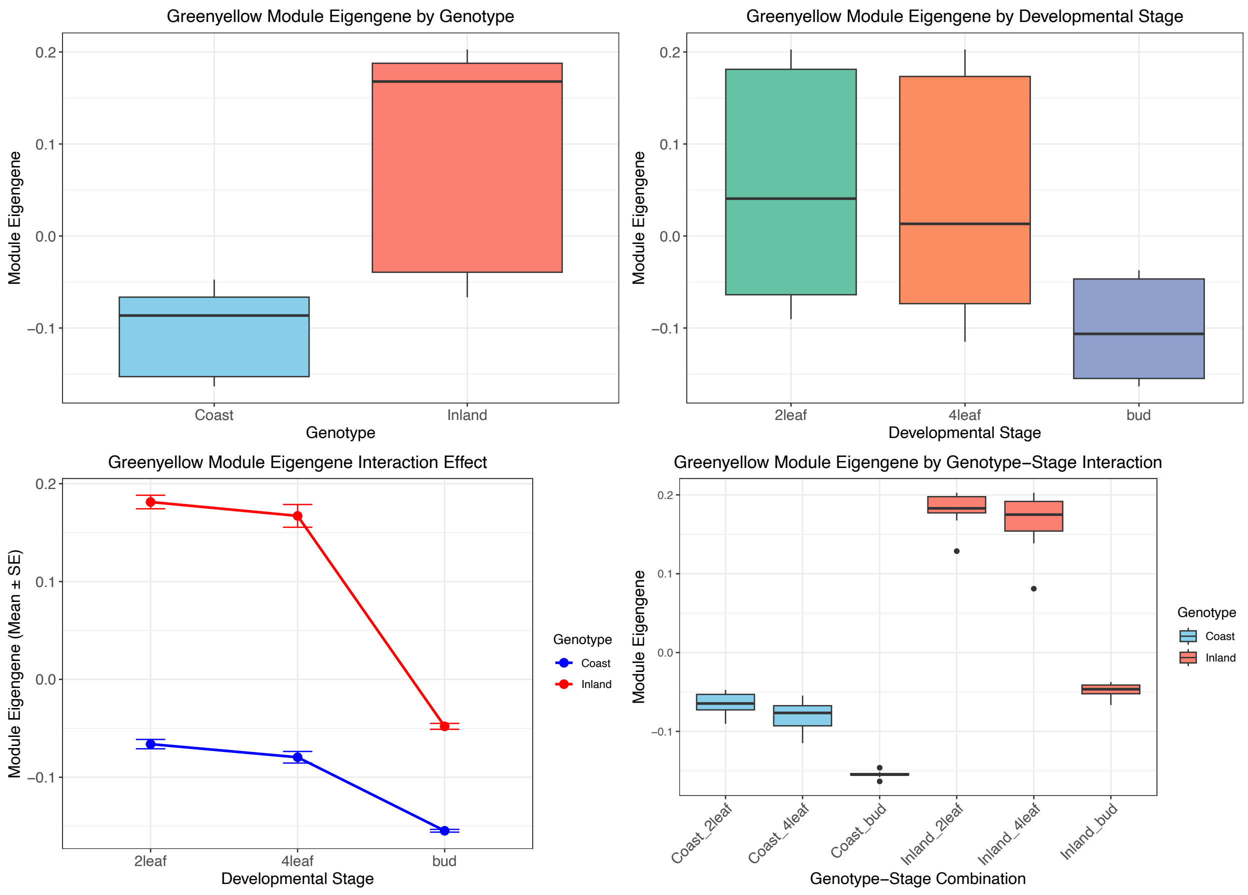

Figure S18. The main effects (genotype and developmental stage) and interactions for the eigengene of the greenyellow module from the WGCNA analysis.

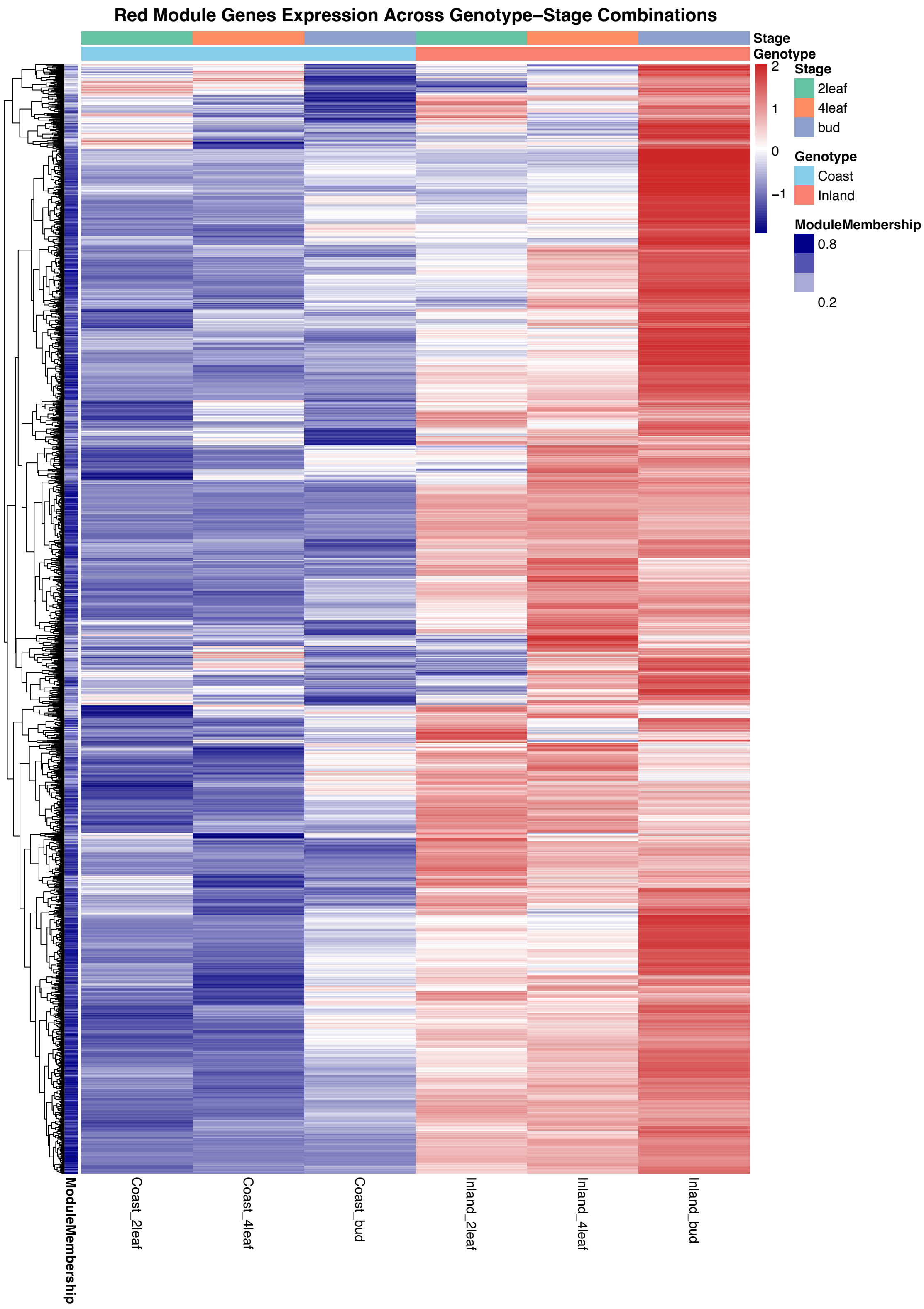

Figure S19. Heatmap of the expression of genes within the red module across genotypes and developmental stages.

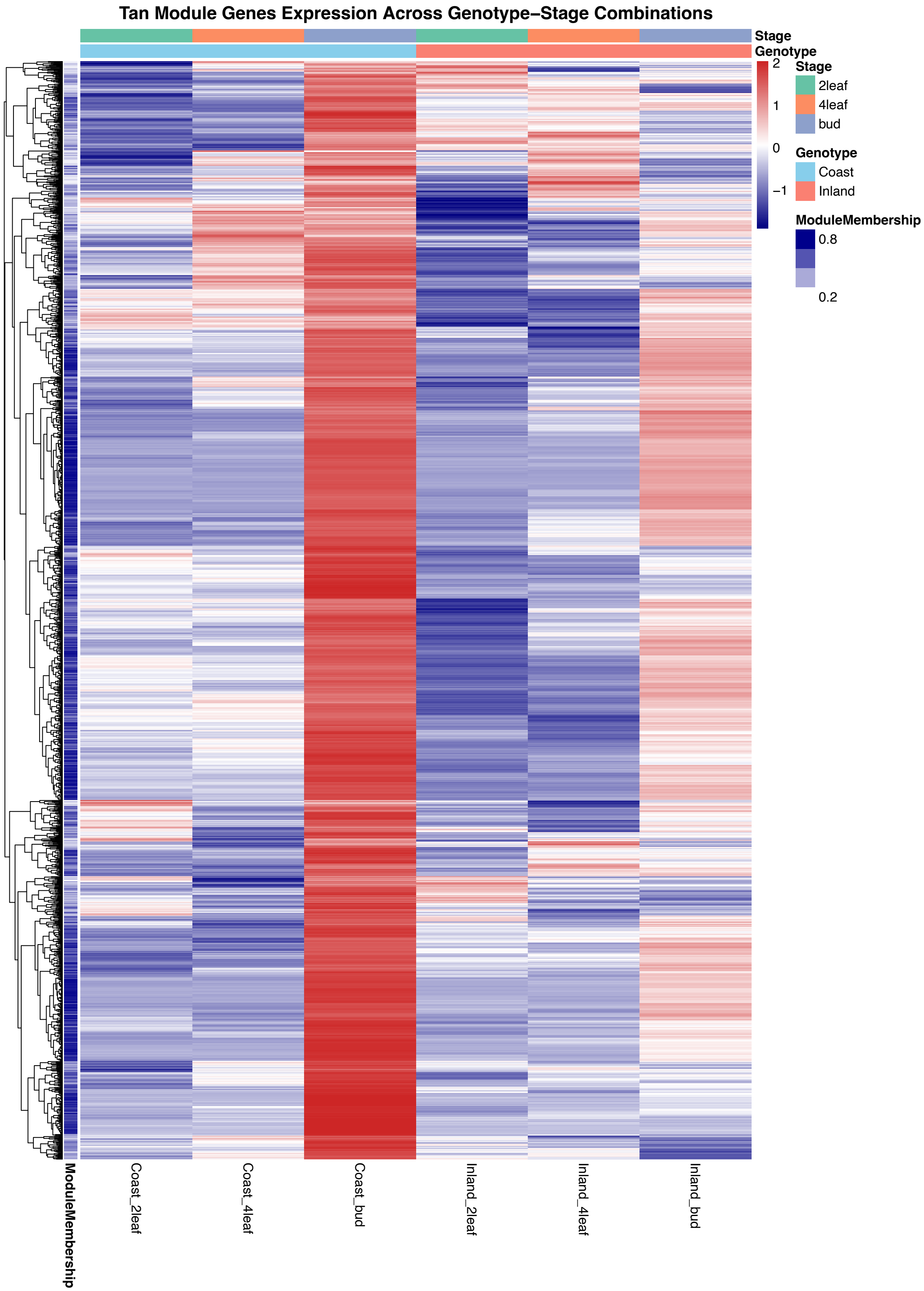

Figure S20. Heatmap of the expression of genes within the tan module across genotypes and developmental stages.

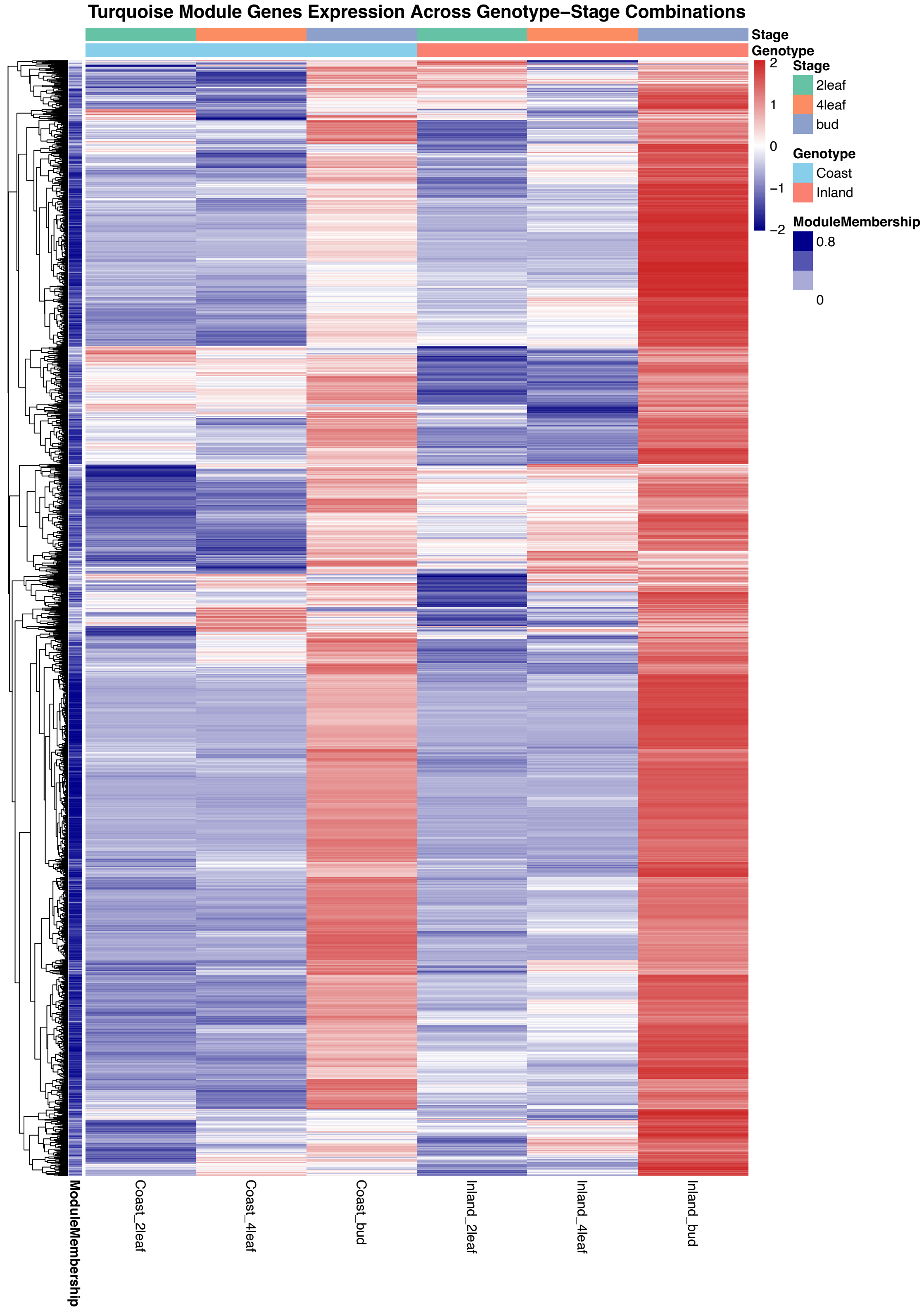

Figure S21. Heatmap of the expression of genes within the turquoise module across genotypes and developmental stages.

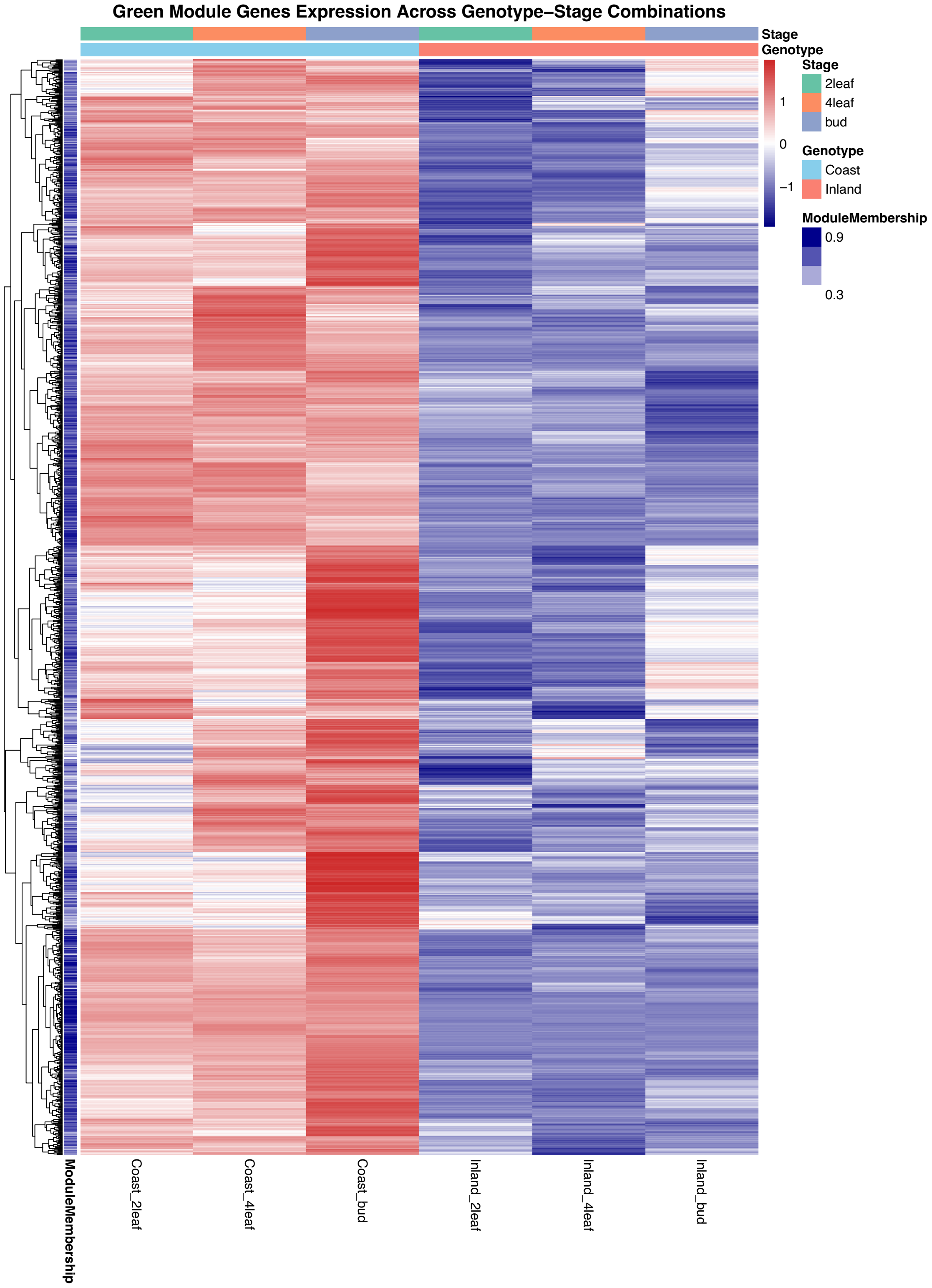

Figure S22. Heatmap of the expression of genes within the green module across genotypes and developmental stages.

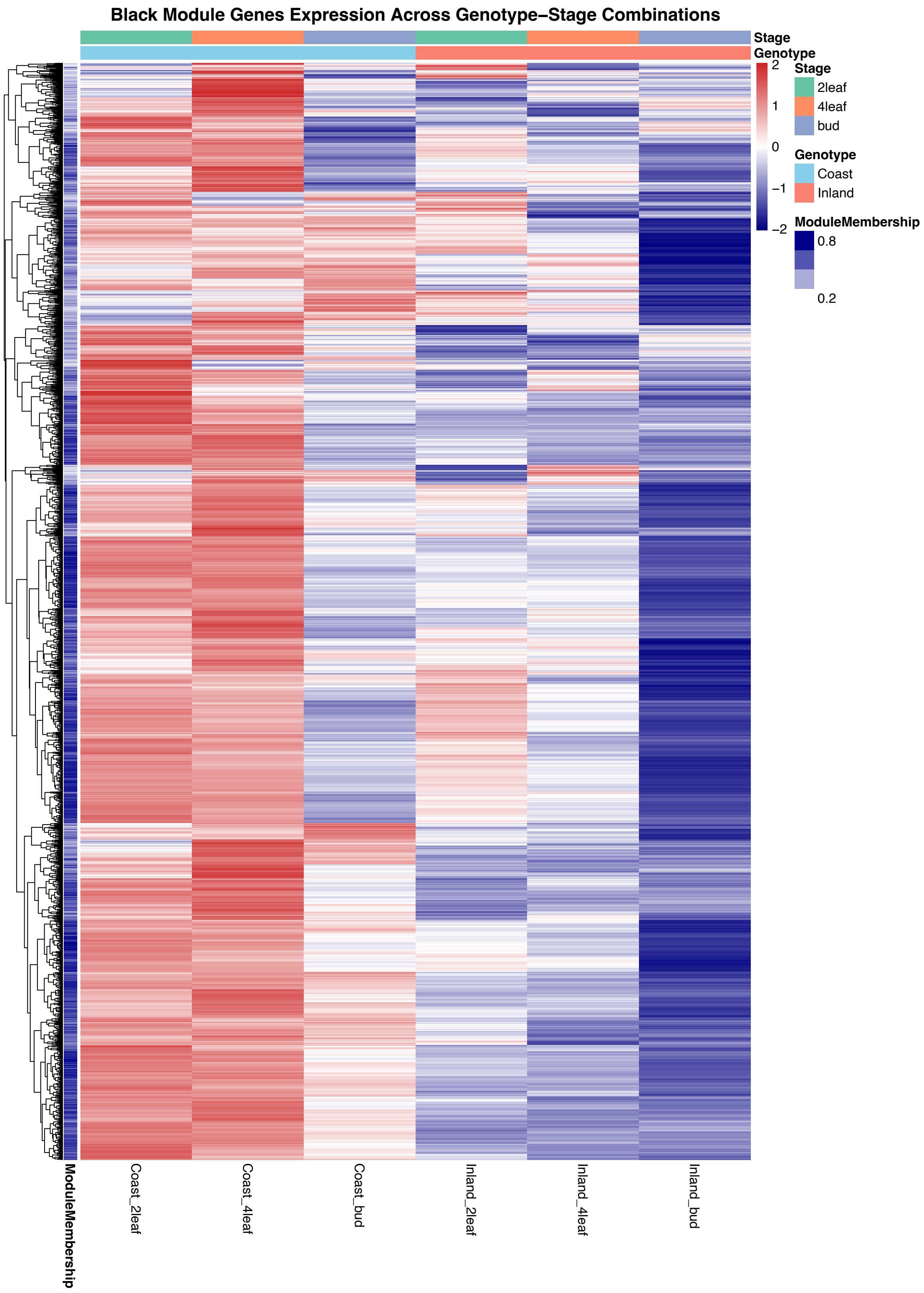

Figure S23. Heatmap of the expression of genes within the black module across genotypes and developmental stages.

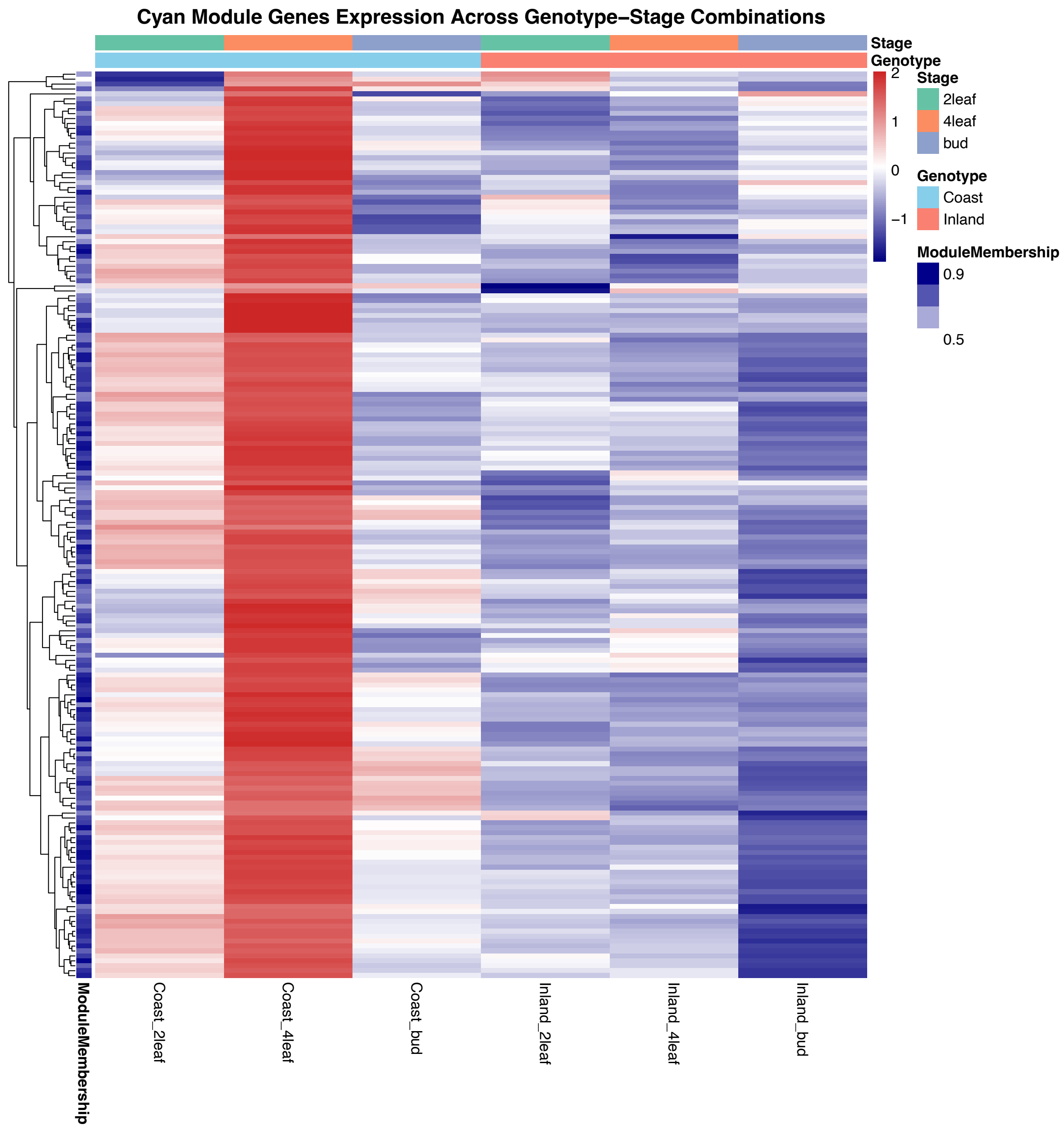

Figure S24. Heatmap of the expression of genes within the cyan module across genotypes and developmental stages.

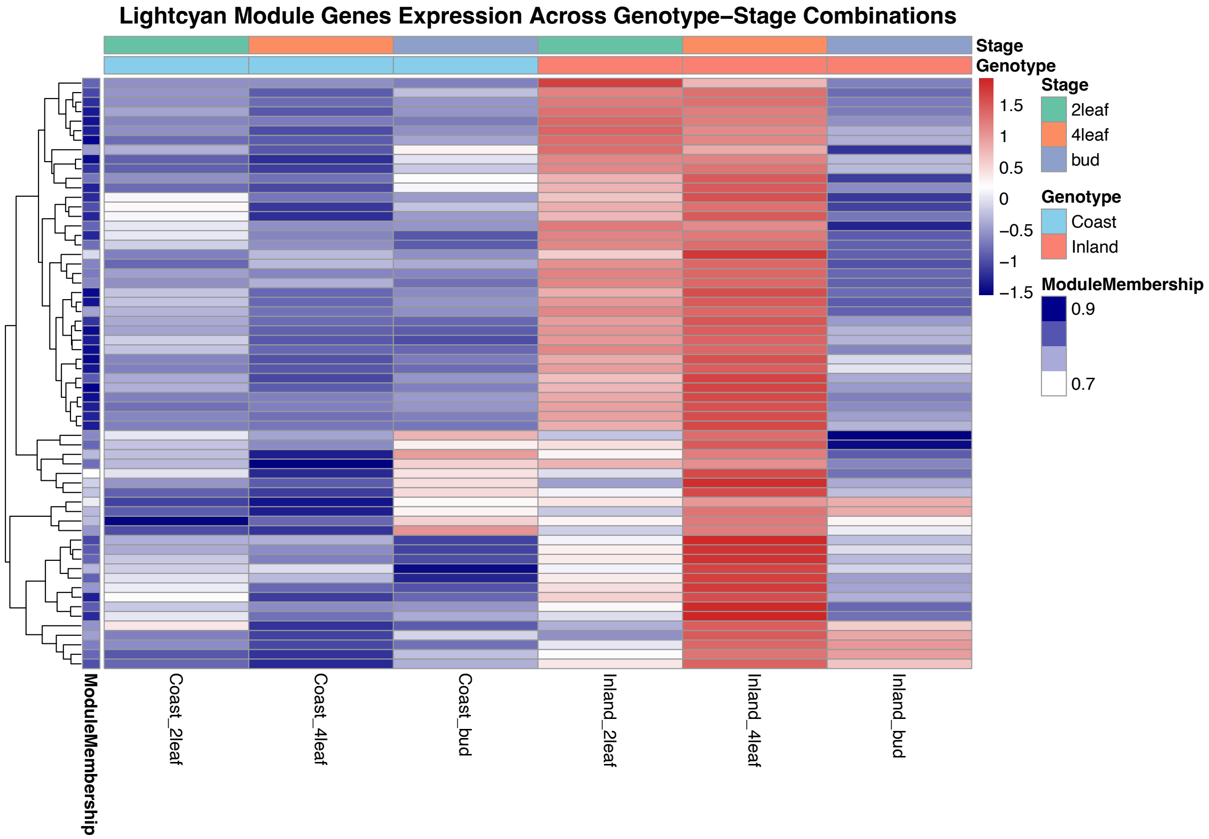

Figure S25. Heatmap of the expression of genes within the lightcyan module across genotypes and developmental stages.

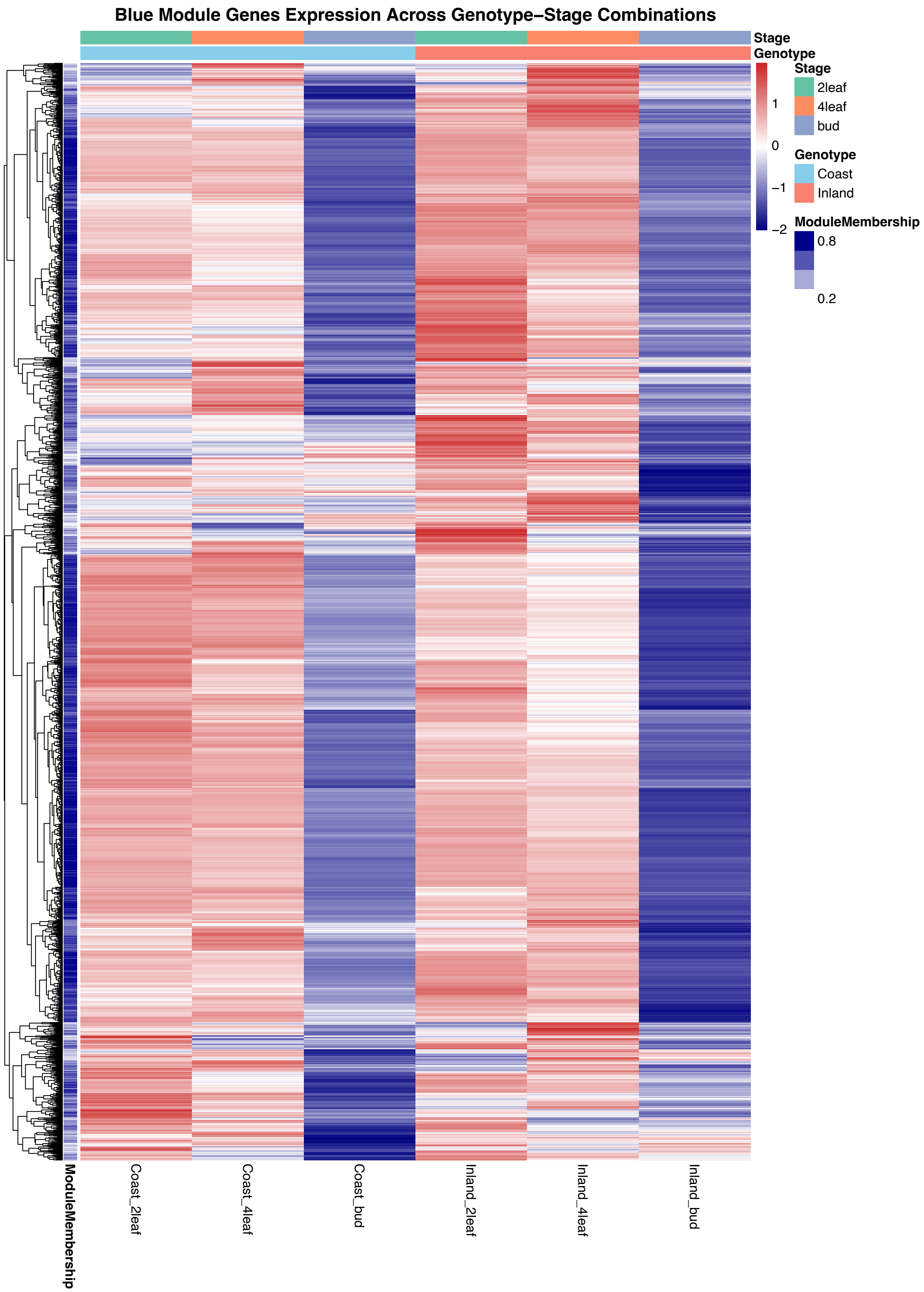

Figure S26. Heatmap of the expression of genes within the blue module across genotypes and developmental stages.

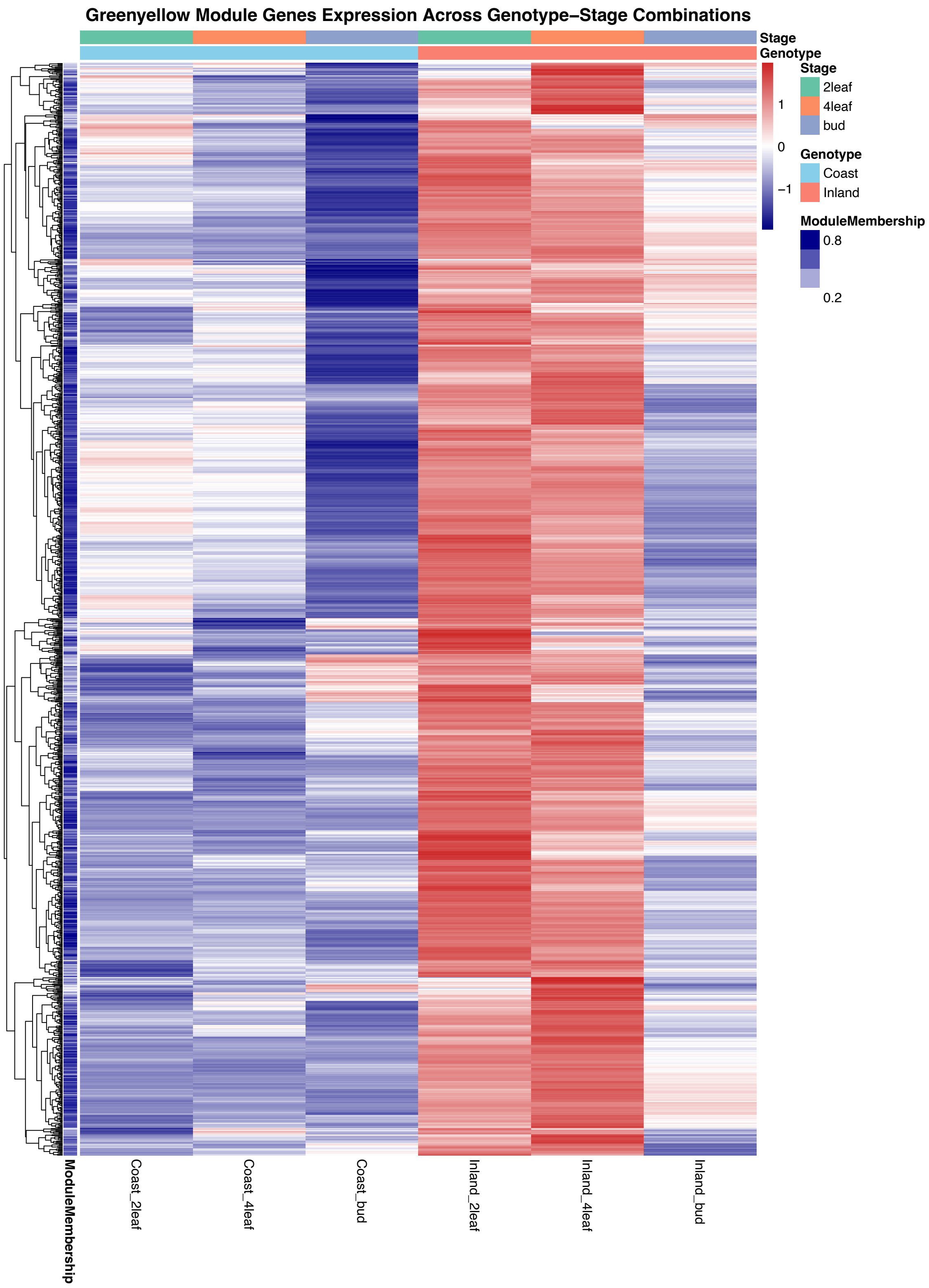

Figure S27. Heatmap of the expression of genes within the greenyellow module across genotypes and developmental stages.
